# The complete chloroplast genome sequence of *Melica scabrosa* (Poaceae)

**DOI:** 10.1080/23802359.2019.1660258

**Published:** 2019-09-06

**Authors:** Jiaojun Yu, Fei Zhao, Shisheng Li, Yuanping Fang, Jun Xiang, Hongjin Dong

**Affiliations:** a Hubei Key Laboratory of Economic Forest Germplasm Improvement and Resources Comprehensive Utilization, Huanggang Normal University, Huanggang, China;; b Hubei Collaborative Innovation Center for the Characteristic Resources Exploitation of Dabie Mountains, Huanggang, China;; c Key Laboratory for Plant Diversity and Biogeography of East Asia, Kunming Institute of Botany, Chinese Academy of Sciences, Kunming, China

**Keywords:** *Melica*, complete chloroplast genome, Poaceae, phylogeny

## Abstract

*Melica scabrosa* Trin. is an important forage grass of Poaceae, wildly distributed in the Northeast Asia to Qinghai-Xizang Plateau. The complete chloroplast genome sequence of *M. scabrosa* was obtained by de novo assembly using whole genome sequence data. The chloroplast genome is 134,889 bp in length, containing 80,560 bp in a large single copy (LSC), 12,706 bp in a small single copy (SSC) and 20,810 bp in a pair of inverted repeats (IRs). A total of 129 genes including 83 protein-coding genes and 38 structural RNA genes were identified. Phylogenetic analysis represented close relationship among *Melica* species. This chloroplast genome sequencing offers a useful resource for future genetics and phylogenetic studies.

*Melica* L. is a genus of the tribe Meliceae in Pooideae, Poaceae (Soreng et al. 2015), comprised by ca. 90 species and distributed in temperate and subtropical regions of the world (Chen et al. 2006). *Melica scabrosa* is a common element of the grassland, wildly distributed in the Northeast Asia to Qinghai-Xizang Plateau and used as an important forage for livestock.

In present study, the completed chloroplast genome sequence of *M. scabrosa* is reported contributing for better understanding its evolution and population genetics, and also providing significant information for the phylogeny of Poaceae.

Genomic DNA was extracted from fresh leaves of *M. scabrosa* from Wujiashan, Yingshan, Hubei, China (115°47′55.77″E, 31°05′32.68″N, 900 m; *Dong* et al.*-HGNU-0039*, 2018-11-2; HTGC), the total genomic DNA was isolated according to a modified CTAB method (Doyle and Doyle 1987). Total genome DNA of *M. scabrosa* was sequenced by Illumina Hiseq 2500 Sequencing System (Illumina, Hayward, CA) to construct the shotgun library and assembled through the NOVOPlasty software (Dierckxsens et al. 2017), with *M. subulata* (GenBank: KM974743) as the reference. The low quality sequences were filtered out Using CLC Genomics Workbench v8.0 (CLC Bio, Aarhus, Denmark). The complete chloroplast genome of *M. scabrosa* was annotated using Geneious ver. 10.1 (http://www.geneious.com, Kearse et al. 2012) and online program Chloroplast Genome Annotation, Visualization, Analysis, and GenBank Submission (CPGAVAS) (Institute of Medicinal Plant Development, Chinese Academy of Medical Sciences and Peking Union Medical College, Beijing, China) (Liu et al. 2012) and then submitted to GenBank (accession no. MN091803). Finally, a physical map of the genome was drawn by using the online program Organelle Genome DRAW (OGDRAW) (Lohse et al. 2013).

The size of chloroplast genome of *M. scabrosa* is 134,889 bp, including a large single-copy (LSC) region of 80,560 bp and a small single-copy (SSC) region of 12,706 bp separated by a pair identical inverted repeat regions (IRs) of 20,810 bp each. A total of 129 genes were successfully annotated containing 83 protein-coding genes, 38 tRNA genes and 8 rRNA genes. GC content of the whole genome, IRs, LSC and SSC regions are 38.5%, 44.0%, 36.6% and 32.6%, respectively. GC content of IRs region is the highest. 14 genes contain one intron, while 3 genes have two introns. The complete chloroplast genome sequence of *M. scabrosa* and other species from Poaceae were used to construct phylogenetic tree (Figure 1). A neighbour-joining (NJ) tree was performed with Mega 6.0 (Tamura et al. 2013) using 1000 bootstrap replicates. The result shows the difference of *M. scabrosa* from Pooideae, which is consistent with previous molecular results (Saarela et al. 2015).

**Figure 1.**
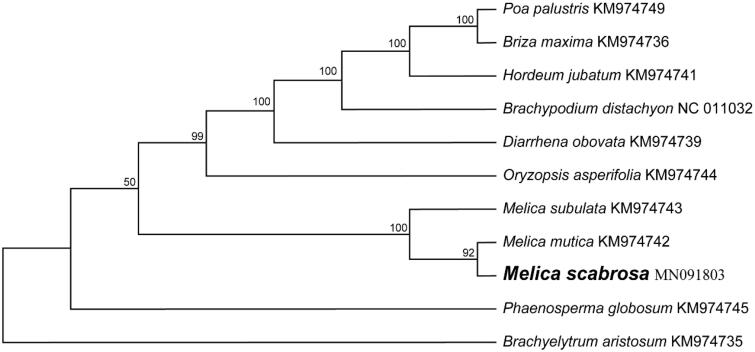
Maximum-likelihood phylogenetic tree of *Melica scabrosa* with 10 other Poaceae species based on neighbor-joining (NJ) analysis of the whole chloroplast sequences. The bootstrap values were based on 500 resamplings, and are indicated next to the branches.
